# Cannabis and cannabinoids for mental health indications: evidence of effect and adverse events

**DOI:** 10.1192/j.eurpsy.2023.101

**Published:** 2023-07-19

**Authors:** J. G. Bramness

**Affiliations:** Norwegian Institute of Public Health, Oslo, Norway

## Abstract

**Abstract:**

Cannabis and cannabinoids have been marketed and sold for a variety of different psychiatric conditions like e.g., anxiety, sleep problems, ADHD, PTSD, and even psychosis. Some of these indications may be reasonable, but for some a more conservative approach should be upheld. There are quite a few open studies and case reports on effects, while larger blinded RCTs either fail to find these effects or are lacking. The lecture aims at presenting the most recent evidence for the use of cannabis and cannabinoids for psychiatric indications, alongside a presentation of adverse effects.

**Disclosure of Interest:**

None Declared

